# Malpigmentation of Common Sole (*Solea solea*) during Metamorphosis Is Associated with Differential Synaptic-Related Gene Expression

**DOI:** 10.3390/ani11082273

**Published:** 2021-08-01

**Authors:** Menelaos Kavouras, Emmanouil E. Malandrakis, Ewout Blom, Kyriaki Tsilika, Theodoros Danis, Panagiota Panagiotaki, Athanasios Exadactylos

**Affiliations:** 1 Department of Ichthyology and Aquatic Environment, School of Agricultural Sciences, University of Thessaly, Fytokou Str., 38446 Volos, Greece; emalandrak@gmail.com (E.E.M.); danis_theo@hotmail.com (T.D.); ppanag@uth.gr (P.P.); 2 Department of Animal Science, School of Animal Biosciences, Agricultural University of Athens, Iera Odos 75, 11855 Athens, Greece; 3 Wageningen Marine Research, Wageningen University & Research, P.O. Box 67, 1970 AB IJmuiden, The Netherlands; ewout.blom@wur.nl; 4 Department of Economics, University of Thessaly, 28hs Octovriou 78, 38333 Volos, Greece; ktsilika@uth.gr

**Keywords:** *Solea solea*, next-generation sequencing, pigmentation, ambicoloration, ion channels, synapses

## Abstract

**Simple Summary:**

Common sole (*Solea solea*) is an important species for the aquaculture industry. Defects in pigmentation of the species are very common in farmed conditions. Differences in gene expression between normally pigmented juveniles and those that present both sides full pigmented, ocular and blind, were investigated. Differentially expressed transcripts were functionally annotated, and gene ontology was carried out. The results indicated that ambicolorated juveniles showed a significant upregulation of genes involved in the signal transmission at the synaptic level and regulation of ion channels, affecting the plasticity and the development of the synapses, as well as the transmission of signals or ions through channels.

**Abstract:**

In farmed flatfish, such as common sole, color disturbances are common. Dyschromia is a general term that includes the color defects on the blind and ocular sides of the fish. The purpose was to examine the difference in gene expression between normal pigmented and juveniles who present ambicoloration. The analysis was carried out with next-generation sequencing techniques and de novo assembly of the transcriptome. Transcripts that showed significant differences (FDR < 0.05) in the expression between the two groups, were related to those of zebrafish (*Danio rerio*), functionally identified, and classified into categories of the gene ontology. The results revealed that ambicolorated juveniles exhibit a divergent function, mainly of the central nervous system at the synaptic level, as well as the ionic channels. The close association of chromophore cells with the growth of nerve cells and the nervous system was recorded. The pathway, glutamate binding–activation of AMPA and NMDA receptors–long-term stimulation of postsynaptic potential–LTP (long term potentiation)–plasticity of synapses, appears to be affected. In addition, the development of synapses also seems to be affected by the interaction of the LGI (leucine-rich glioma inactivated) protein family with the ADAM (a disintegrin and metalloprotease) ones.

## 1. Introduction

The remarkable color diversity observed in fish comprises a variety of simple and more complex chromatic patterns with more and less intense colors. The mild to dark coloration of species aims to conceal and make them difficult to track by emulating the shades of the seafloor environment [1]. This category includes flatfish and, particularly, the common sole.

After metamorphosis, the common sole displays a lower, light (white) colored “blind” side, and a superior “ocular” side, with dark, amber coloration. The observed chromatic asymmetry appears to be modulated and synchronized during the step of migrating one eye to the upper-ocular side [2].

In amniotes, adult pigmentation depends on the programmed differentiation of melanocytes containing melanin. These cells contribute to the shading of the stratum corneum and consequently of the skin, hair, and feathers. On the contrary, teleosts and ectodermal vertebrates developed a variety of pigment cells, also known as chromophores, which intracellularly contain a variety of pigments.

The normal development of pigmentation depends on both general mechanisms and the tissue environment that differentiate the pigment cells [3,4]. These cell types originate from the neural crest and migrate to the entire embryo during ontogenesis. In flatfish, the pigmentation establishment involves two stages: (1) during the embryonic and larval developmental stages, the precursor pigment cells (stem cells) migrate to both sides, blind and ocular, which then differentiate into larval melanophores, (2) during the metamorphosis stage the larval melanophore cells disappear and new mature chromophore cells are formed and installed. This time, mature melanophores are found only on the ocular side. Chromatic aberrations appear to occur during the second stage [3,5]. This chromatic asymmetry that occurs after the metamorphosis stage is supposed to be due to the asymmetric presence and expression of the factors that control the differentiation of precursor chromophores, their survival, and propagation. The abnormal function of these mechanisms may lead to the occurrence of chromatic abnormalities, such as pseudo-albinism and hypermelanosis [4]. The variation in the pigmentation pattern observed is due to quantitative variations in either the density or morphology of pigment cells, especially melanophores. In light-colored skins, the melanophores are scattered and shrunken, while on dark surfaces, they are more common and larger [3].

In winter flounder (*Pleuronectes americanus*), the melanophore response during the pigmentation process is nerve-controlled, while the xanthophores are controlled by the pituitary gland. The α-adrenergic receptors mediate the concentration of melanophore cells as opposed to β2-adrenergic receptors mediating their diffusion and dispersion [1]. Following metamorphosis, most flatfish, such as common sole, have a light-colored (bottom-blind) side rich in iridescent cells and a darker top-ocular side that comprises melanophores, xanthophores, erythrophores, and iridophores (on the skin). The ocular side also contains a variety of patterns and colors that are characteristic of each species [1]. This dorsoventral chromatic polarity is achieved by the melanization inhibitor factor (MIF), which inhibits the differentiation of melanoblasts and promotes the propagation of the iridophore cells in the abdomen. The protein Agouti-Signaling Protein 1 (ASIP1) has recently been discovered in fish as the corresponding MIF [4].

In farmed flatfish, such as sole, coloring disorders are common and are described by the term “malpigmentation” [3,4,5,6,7,8,9,10,11]. Dyschromia is a general term that includes the color defects on the blind and ocular side of the fish, which are subdivided into three categories: (1) full or partial alpinism (pseudo-albinism or hypoplasia) related to the ocular side, (2) ambicoloration, when the blind side displays the same pigmentation as the ocular side, and (3) staining or hypermelanosis when parts of the blind side appear to be similar to the ocular one [3].

Deviation from normal pigmentation has an impact on the survival of the species in the marine environment, as well as on the sale price of farmed animals [5]. Anomalies in chromatic patterning are rarely seen in the wild flatfish populations, including common sole [6,12]. A common working hypothesis is that common sole, such as Senegalese (*Solea senegalensis*), are species that present complexity during the stages of embryogenesis and metamorphosis and difficulties in weaning associated with chromatic and skeletal abnormalities [13,14,15]. It is assumed that both the coloring and the morphological characteristics are determined early by the so-called “coloring or pigmentation window”, which is defined as the period of ontogenesis where nutritional factors, such as polyunsaturated fatty acids, docosahexaenoic acid (DHA), eicosapentaenoic acid (EPA), arachidonic acid (ARA), and Vitamin A play a key role in coloring [5,16].

Another hypothesis would be the role of stress and rearing conditions in the early stages of life. The degree of coloration of the blind side (overspill or blot) has been reported to be related to environmental and external factors, such as the color of the tanks [7]. The white background seems to stimulate the melanin concentrating hormone (MCH), an endocrine hypothalamo-pituitary peptide, which causes pale body coloration [7,17]. Instead, high fish density seems to lead to hypermelanosis of the blind side in the Olive flounder or Japanese flounder (*Paralichthys olivaceus*) up to 95%. Therefore, light-colored adaptation to environmental conditions is induced by the high concentration of the hormone MCH, while dark-colored inhibits it [17].

It has been confirmed that diet with high levels of ARA or ARA/EPA in the early stages of the Senegalese larvae, before metamorphosis, not only is associated with abnormal coloring but also affects the melanophore density and skin complexion in both normal and pseudo-albino subjects. The main causes are epigenetic factors [18]. In Senegalese sole, the high levels of ARA are also associated with an increase in type II prostaglandins (PGE2), pseudo-albinism, and cranial malformations, mainly related to ocular disorders [19]. In common sole, paleness occurred at the larval stage in subjects who did not receive ARA as a dietary supplement. Vitamin A is involved in numerous biological functions [5].

Stress conditions and endocrine disorders are also related to the metabolic pathways involved in pigmentation. Hypermelanosis in flatfish has been linked to an elevated concentration of cortisol, indicating that stress during rearing conditions is a possible factor for pigment abnormalities [20]. In Nile tilapia (*Oreochromis niloticus*), adaptation in a dark-colored environment activates melanocyte-stimulating hormone (α-MSH). Its long-term administration stimulates the development of melanocytes. The abnormal regulation of MCH and α-MSH hormones and their receptors may also cause pigmented spots or hypermelanosis of the blind side [7]. Endocrine disorders are related to pigmentation through thyroid hormone fluctuation in adult pigment pattern development in zebrafish [21]. In Japanese flounder, overexpression of thyroid hormone is coupled with a high occurrence of albinism due to inhibition of melanophore proliferation [21]. Other endocrine disorders are related to the deregulation of α-MSH production in summer flounder (*Paralichthys dentatus*) [5,21]. The light-brain-pituitary axis is affected by environmental factors that alter physiological α-MSH production and secretion [21].

Ambicoloration and albinism are characterized as high-level design errors associated with bilateral symmetry, while staining and hypomelanosis mainly represent local disturbances. Ambicoloration is a developmental phenomenon: it occurs at a specific point of ontogenesis (during the metamorphosis), is irreversible, comprises all structures of skin and epidermis, and depends on melanophore density. Ambicoloration is influenced by factors that affect the whole-body design and not only locally. In contrast, staining also occurs in older individuals, is mainly influenced by environmental factors, and in some cases is reversible [3]. Noteworthy is the fact that there have not been observed individuals who exhibit both hyper- and hypomelanosis, i.e., simultaneous pigmentation abnormalities on both sides, supporting the assumption that the same mechanism controls the pigmentation on both sides [9].

The pigmentation gradient of the blind side (hypermelanosis or staining) has been reported to be related to environmental and external factors, such as the color of the tanks [7]. The expression level of melanin concentrating hormone (MCH) is influenced by the background color [17]. A white background seems to stimulate the hormone (MCH) that causes pale body coloration. To date, it is suggested that the incorrect regulation of MCH and α-MSH hormones and their receptors may be the cause of staining or hypermelanosis of the blind side [7].

It is clear that further studies into specific gene regulation and pigment abnormalities are needed. The purpose of this work was to investigate the differences in gene transcription levels between normal and ambicolorated individuals, with their blind side full-colored, and also identify biological-molecular pathways that affect the pigmentation process in common sole.

## 2. Materials and Methods

### 2.1. Sampling

The treatment of juveniles was in line with the EU Directive 2010/63/EU on the protection of animals used for experimental and other scientific purposes [22]. Broodstock breeding, spawning, and rearing of larvae and juveniles of common sole took place at IMARES (now WMR), Ijmuiden, The Netherlands. The examined larvae originated from a wild-caught broodstock, accordingly acclimatized for experimental protocols. Rearing and spawning conditions were set according to previously published protocols [15,23,24]. One could safely argue that different strains of Dover sole originated from different genetic sources. A positive heterozygosity fitness correlation (HFC) suggests that special precautions should be taken into account whenever different genetic structure patterns emerge because of various flatfish broodstock geographical origin and year class samples [14,25,26,27]. After hatching, the larvae were randomized into three groups and transferred to separate 300 L, cubic-shaped, light-brown colored polyester tanks. Seawater was recycled through pumps with an average renewal rate of 4 h (3 h after the 7th day) in a special filtration system, including mechanical, biological, chemical, and UV filters. The temperature was set at 12 °C, increased by 1 °C per day until the 6th day, and then held constant at 17 °C while the salinity was at 34.3 psu and the pH set at 7.7. On the fourth day post-hatching (dph), larvae began feeding with artemia once a day, while from the 8th dph and onward, they were fed with enriched one food, twice a day. An artificial lighting regime, 12 h day and 12 h night, was applied from the 7th dph onward. The salinity gradually declined to 26 psu by the 15th dph. The sampling of the juveniles took place on the 24th dph, at the time when individuals with ambicoloration (total coloration of the blind side) fully appeared. Three normal pigmented individuals and three ambicolorated ones, standard length 9.3–9.5 mm, were randomly harvested from each tank, placed on ice, washed with sterile water, and set in separate vials (Falcon 50 mL) complemented with RNA later. They were stored at −24 °C until the extraction and isolation of total RNA.

### 2.2. RNA Extraction and Sequencing

Three normal pigmented samples and three ambicolorated ones were used for total RNA extraction, using the Nucleospin TriPrep reagent series (Macherey-Nagel, Germany), following the manufacturer’s instructions. Each sample consisted of 10 μL homogenized tissue of three juveniles. In particular, three juveniles, after washing with distilled water, were placed in Eppendorf vials and homogenized manually, using a mechanically rotating plunger. A total of 10 μL of homogenized tissue was used to extract the total RNA. In total, six samples (three with normal pigmentation and three with ambicoloration) were sent to ServiceXS/GenomeScan B.V., Leiden, The Netherlands, for libraries construction and sequencing. The “NEBNext Ultra Directional RNA Library Prep Kit for Illumina” reagent set (NEB # E7420S/L) was used according to the manufacturer’s instructions for further processing. The isolation of mRNA from total RNA was performed using oligo-dT magnetic beads. Following mRNA fragmentation, cDNAs were synthesized and ligated to sequencing adapters. The quality and enrichment of the processing products were assessed with a BioAnalyzer Fragment Analyzer. The size of the products was in line with the expected size distribution (between 300 and 500 bp). The sequencing was performed with Illumina cBot and HiSeq 2500 (San Diego, CA, USA), according to the manufacturer’s instructions. The untreated raw data for the six created libraries were deposited in the NCBI Sequence Read Archive (study accession number PRJNA324631). All samples meet the sequencing requirements.

### 2.3. Bioinformatics Analysis

#### 2.3.1. Read Pre-Processing

The sequencing yielded 159,651,407 pair-end reads, with a length of 125 bp. Quality control was performed using the FastQC v. 0.11.3 [28], followed by the succeeding cleaning steps, each of which was again accompanied by quality control: (a) Removing low-quality reads with PRINSEQ v.0.20.4 [29] setting the parameters, quality score, and tail trimming at > 20 and > 5, respectively. (b) Trimming adapters or primers with the cutadupt v.1.7.1 [30]. (c) Re-trimming of low-quality reads, again with the PRINSEQ v.0.20.4 and with setting parameters: -min_len 90, -min_qual_mean 30, entropy -lc_threshold 50, and -trim_tail_right/left 5. (d) Step (b). (e) Exclusion of ribosomal RNA by sortMeRNA v.2.0 [31]. Following the aforementioned sequential processing steps, 123,676,932 high-quality pair-ended reads were retained, which were used to assemble the transcript.

#### 2.3.2. Transcriptome Assembly and Annotation

For the transcriptome assembly, all examined reads were used. Trinity v.2.0.6, with a fixed parameter, kmer 25, was used as the assembler of choice [32]. The draft transcriptome was tested for transcripts not supported by the reads. Spurious ones were filtered using the Perl script file, align_and_estimate_abundance.pl, based on the Bowtie2 and RSEM of Trinity. As parameters with optimal results, they were set, --fpkm_cutoff 0.2 and -isopct_cutoff 1. In order to optimize the next steps of the analysis, control and removal of redundant transcripts were performed using the CDHIT software [33]. For their functional annotation, the assessed transcripts were initially “blastxed” (threshold: e = 10^−6^), with the NCBI’s *nr* protein database and consequently “blastned” (threshold: e = 10^−9^), with the nucleotide database, resulting from the mRNA collection of Actinopterygii: Mexican tetra (*Astyanax mexicanus*)*,* tongue sole (*Cynoglossus semilaevis*)*,* Atlantic cod (*Gadus morhua*)*,* threespine sticklebacks (*Gasterosteus aculeatus*)*,* spotted gar (*Lepisosteus oculatus*)*,* Nile tilapia, medaka (*Oryzias latipes*)*,* amazon molly (*Poecillia formosa*)*,* Japanese pufferfish (*Takifugu rubripes*)*,* spotted green pufferfish (*Tetraodon nigroviridis*)*,* common sole, Senegalese sole, and Southern platyfish (*Xiphophorus maculatus*).

The completeness and the integrity of the assemblies were evaluated by the CEGMA v.2.5 [34]. The transcripts with low values ε (ε < 10^−30^) and high overlay (>70%) were selected to compose the reference transcriptome. For the estimation of gene expression, the reads were aligned with the reference transcriptome (two treatments, triplicates) by utilizing Bowtie2, and the relative gene expression was evaluated with the DESeq2 [35]. Expression value: |log_2_FC |> 1, and significance, FDR (Benjamini–Hochberg false discovery rate): <0.05 were defined as the threshold of differential expression. Transcripts with a difference in expression were mapped with those of zebrafish (blastn, e < 10^−9^) and annotated. This allowed for the classification of each differentially expressed transcript to the corresponding gene ontology class (GO) by using DAVID v6.8 fisher exact test [36]. Furthermore, overrepresentation analysis was carried out with Cytoscape v.3.2.1. and the BINGO v.3.0.3. [37].

## 3. Results

### 3.1. Transcriptome Assembly

**Figure 1:**
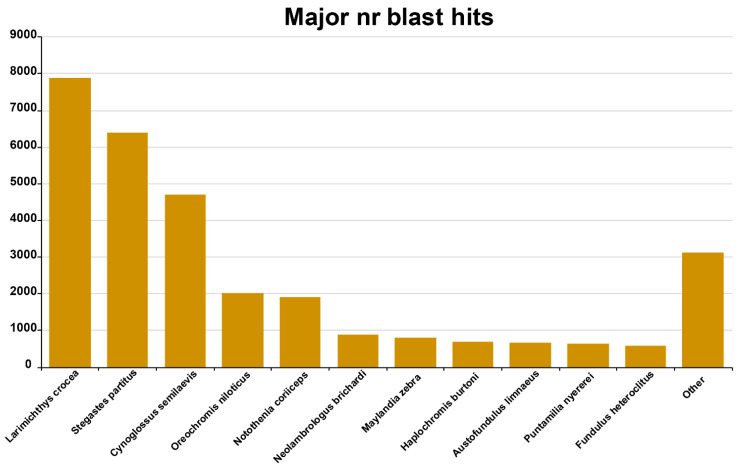
Major nr blast hits. *X*-axis: number of transcripts blasted. *Y*-axis: name of species.

After the meticulous and rigorous quality processing οf 159,651,407 pair ends reads with an average length of 125 bp, 123,676,932 pair-end reads of a high-quality, with a length of 100–110 bp, were obtained and analyzed. These were used for de novo transcriptome assembly, which produced 381,106 unique contigs. Transcripts not supported by reads were removed by TRINITY’s Bowtie2 and RSEM, and a total of 142,918 contigs consequently remained. The implementation of the CD-HIT reduced the number of contigs to 85,932, presenting a total of 70,243,227 nucleotides, N50 value 1563 bp, and a maximum transcript length of 29,649 bp. Transcriptome assemblies were evaluated by CEGMA v.2. The analysis showed that 214 of 248 core proteins (86.29%) were found to be complete. On average, two orthologs per reference protein were detected, and the percentage of eukaryotic reference genes (CEGs) having more than one orthologous gene was 50%. To classify the already tested transcripts (85,932) to the corresponding gene ontology terms (GO), they were blastxed with the NCBI protein database, *nr*, and they were mapped to 56,179 sequences, of which 33,878 were unique for common sole. The unique transcripts were more related to the species, yellow croaker (*Larimichthys crocea*) 7.898 (23.31%), bicolor damselfish (*Stegastes partitus*) 6.406 (18.91%), tongue sole 4.696 (13.86%), Nile tilapia 2.011 (5.94%), black rock cod (*Notolahenia coriiceps*) 1.903 (5.62%), fairy cichlid (*Neolambrologus brichardi*) 884 (2.61%), and zebra mbuna (*Maylandia zebra*) 809 (2.39%), as described in Figure 1 [38].

### 3.2. Gene Expression

In total, 233 transcripts of common sole were recorded to exhibit differential gene expression between the normally pigmented individuals and the ambicolorated ones (log_2_FC1 and FDR < 0.05). Of these, 157 genes were identified in zebrafish. The most significant Reactome database paths detected based on the 157 identified transcripts are shown in Table S1 and Table 1.

**Figure 2:**
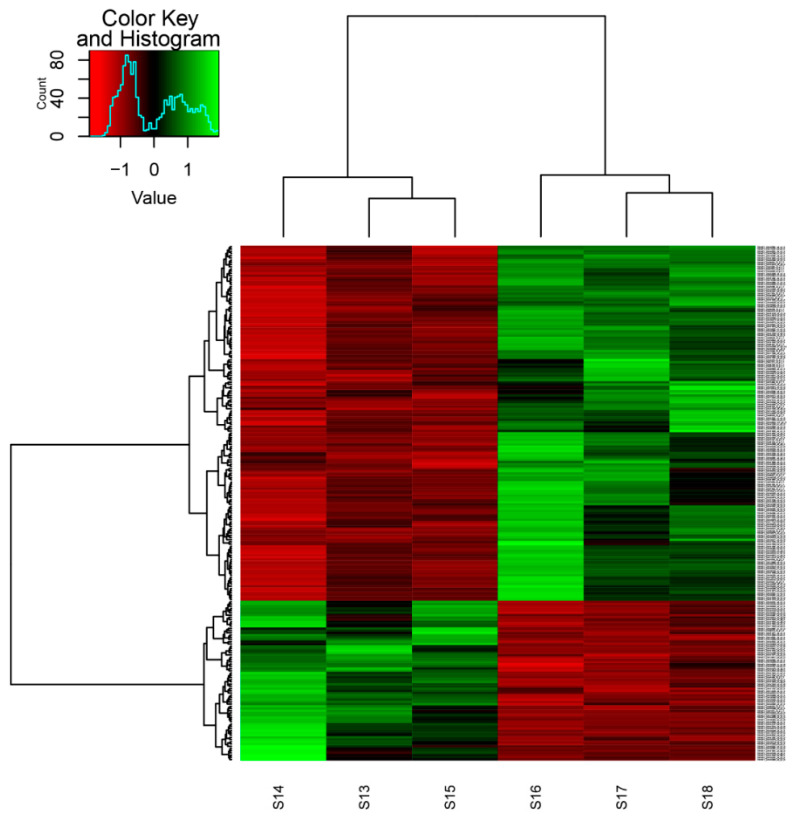
Heat-map illustrating gene expression profiles of transcripts. The *x*-axis represents the six RNAseq libraries from the two groups (S13, S14, S15 normal pigmented, S16, S17, S18 ambicolorated), and the *y*-axis represents the transcripts clustered according to their expression pattern. Gene expression is color-coded; red represents the down-regulated transcripts and green the upregulated ones.

Out of the 233 differential expressed transcripts of common sole, 161 were upregulated, while 72 were downregulated. Accordingly, out of the 157 identified transcripts, 94 were upregulated and 63 downregulated. Table S1 indicates the transcripts with the greatest changes in expression. A more detailed comprehensive view of gene expression patterns between the normally pigmented individuals and the ambicolorated ones is illustrated on a heat map in Figure 2.

**Figure 3:**
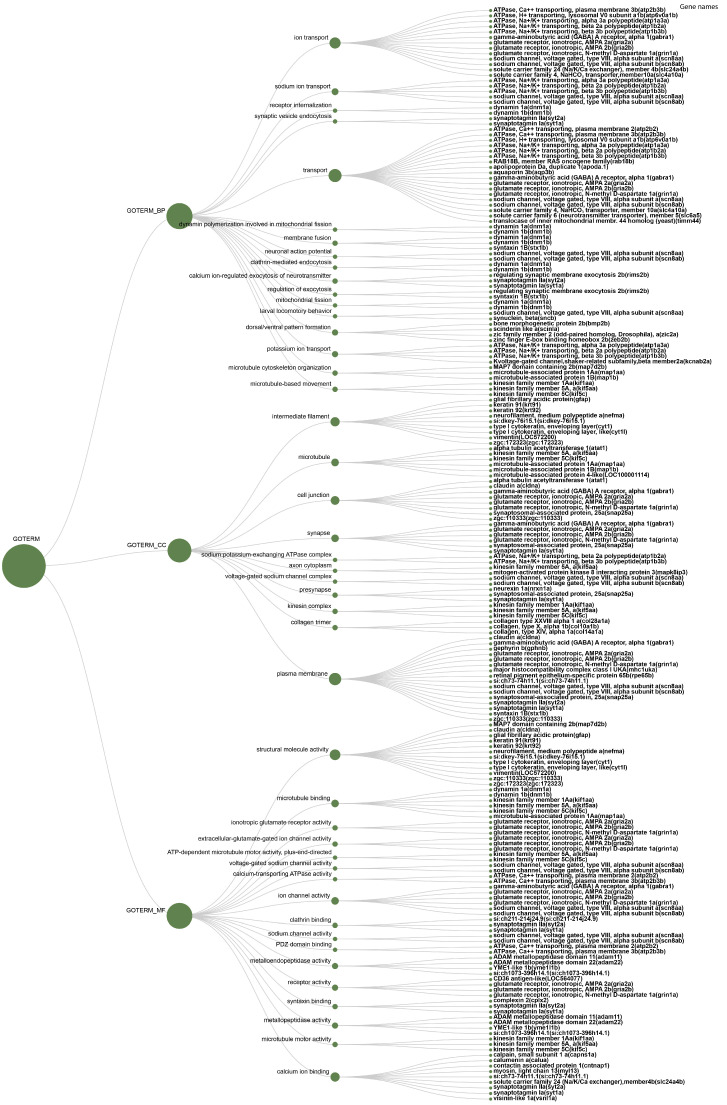
Gene ontology, Biological function (BF), Molecular function (MF), Cellular component (CC). The gene names appear to the left of the figure.

The 157 differentially expressed transcripts, mapped to their ortholog from zebrafish, were ranked in terms of gene ontology categories. Gene ontology enrichment analysis was performed in DAVID v6.8 determined by Fisher Exact test (*p* < 0.05) [36]. The differentially expressed (d.e.) transcripts grouped as biological process, cellular component, and molecular function are presented in Table 2 and Figure 3**.**

## 4. Discussion

Ambicolorated common sole juveniles exhibit differential global gene expression when compared to normally pigmented fish. Differentially expressed genes were mainly involved in the ion transport homeostasis as members of ion channels affecting muscular and cardiac functions, and others involved in the synaptic level at the central nervous system. The formation of the corneal envelope related to the period of embryonic development, barrier malfunctions, and structural defects in the desmosome connections appeared to be affected.

According to the gene ontology enrichment analysis in combination with analysis carried out through the “Reactome” database, from 20 different biological paths, involving the 157 identified gene sequences, 10 of them are associated with the central nervous system, 4 with developmental functions, 3 with muscular and cardiac function, 2 with the transportation of small molecules, and 1 with the communication between cells. Ion channels are also important strains of CNS directly related to neurotransmitters and receptor proteins at the synaptic level. Concerning the nervous system, important roles display the receptors of neurotransmitters and the signal transmission in the postsynaptic area [39]. The axis, glutamic acid-binding–activation of AMPA receptors (α-amino-3-hydroxy-5-methyl-4-isoxazolepropionic acid)–plasticity of synapses, seems to be altered in ambicolorated individuals.

The associated pigmentation ion channels, when activated, increase intracellular Ca^++^ and melanin. Ca^++^ could also control the synthesis and storage of the pigment in melanosomes. Ca^++^ inflow through the melanosome membrane probably regulates the membrane voltage, luminal pH, and could adjust the enzymatic activity. Through a mechanism involving exocytosis and endocytosis, Ca^++^ probably facilitates the transfer of melanin. Similarly, in neurons and endocrine cells, Ca^++^ facilitates the exocytosis of vesicles [40].

Ion transfer not only regulates pigmentation through ionic messengers such as Ca^++^, K^+^, and Na^+^ but also induces a strong cellular signal by varying the membrane potential. The intense and quick changes in the membrane potential are characteristics of stimulated cells that enable the rapid communication between neurons, muscle, and endocrine cells. These variations are also important in relaxed pigment cells for signaling. Similarly with ion messengers, membrane potential could act as a signal favoring the membrane fusion process of intracellular organelles, including melanosomes [40].

In the brain, the transmission of stimulation through synapses is performed by glutamate receptors via activation of both ionotropic and metabotropic receptors. The ionotropic glutamate receptors are divided into three subtypes according to their distinct physiological characteristics and their differences regarding their ligands, (a) the NMDA (N-methyl D-aspartic acid), (b) AMPA, and (c) kainic acid receptors. These receptors are composed of four subunits, GluR1, GluR2, GluR3, and GluR4, which combine to form a quartet [41,42,43]. The signaling of glutamic acid ion channels begins with the influx of ions. Postsynaptic signal propagation includes the commitment of ligand (glutamate) with the AMPA receptor. This interaction provokes the subsequent influx of Na^+^ ions, which causes the depolarization of the membrane. In the resting state, the NMDA receptors remain blocked by Mg^++^ ions. They are activated by the depolarization of the membrane (caused by AMPA receptors) and by their connection with glutamic acid and glycine. The NMDA receptor is permeable to Ca^++^ ions, and its activation leads to the increment of Ca^++^ concentration. This activation, subsequently, leads to the upregulation of AMPA receptors at synapses that induce long-term stimulation of the postsynaptic potential (excitatory postsynaptic potential, EPSP), which is the basis for the establishment of long-term potential (long term potentiation, LTP) [41,42,43]. The long-term potential contributes to synaptic plasticity. The strength of synapses is reinforced either through phosphorylation or by the substitution of the type of receptors. The phosphorylation of AMPA receptors changes their position, strengthens the channel conductivity, and increases the possibility of opening them. Long-term potential causes a decrease of AMPA receptors [43]. The role of glutamate in melanocyte regulation was studied in Nile tilapia. The blockage of AMPA and NMDA receptors provoked a reversible change in melanocyte morphology by altering the organization of actin and tubulin microfilaments and also by downregulating the expression of the melanocyte differentiation and proliferation factor MiTF, probably leading to melanocyte associated disorders [44].

The results depict that both AMPA receptors and receptors containing the GluR2 subunit are affected. The trafficking of receptors carrying the GluR2 subunit is dominated by protein–protein interactions, which are regulated by phosphorylation. Under conditions of intense presynaptic activity, there is a removal of receptors devoid of the GluR2 subunit and their selective integration into the synapses of AMPA receptors containing the GluR2 subunit. In this process of selective AMPA receptor substitution, proteins interacting with C kinase (PICK) and N-ethylmaleimide-sensitive fusion protein (NSF) are involved in the distribution and transportation to the synaptic region [45,46] of the AMPA receptors, which contain the GluR2 subunit. The action of the NSF protein, with ATPase properties, cleaves the PICK protein from the GluR2 subunit while retaining the receptors in the cell membrane (synapsis). Note that receptors carrying the GluR2 subunit are continuously recycled from cellular endoplasms at synapses and vice versa. In this process, the proteins PICK, and glutamate receptor-interacting protein (GRIP) compete to bind to the GluR2 subunit at the C-terminus, as well as PKC [47].

The results of our study appear to be directly related to such activation of AMPA receptors. The receptors are functionally permeable or impermeable to the Ca^++^ ions, depending on the subunits’ components. Permeability to Ca^++^ is determined by the presence of the GluR2 subunit, which undergoes post-transcriptional modification by changing the glutamine (Q) residue in the pore to arginine (R). AMPA receptors permeable to Ca^++^ also allow the passage of sodium ions (Na^+^). Commonly, glutaminergic neurons contain receptors impermeable to Ca^++^.

As mentioned above, NMDA receptors are a class of glutamic acid ionotropic receptors that are activated by the glutamic acid agonist, N-methyl-D-aspartic acid. Their activation involves the opening of ionic pores that allow the influx of calcium ions. This class of receptors is at the heart of the changes regarding synaptic strength and synaptic plasticity and is also associated with learning and memory [48,49].

Data yields Ras protein activation as a result of stimulation of NMDA receptors and Ca^++^ influx through the Ras Guanine nucleotide releasing factor (RasGRF). The binding of RasGRF to the Ca^++^/calmodulin complex in the presence of high concentrations of Ca^++^ leads to the activation of the Ras protein, which exchanges GDP (guanosine diphosphate) in GTP (guanosine triphosphate) [49,50].

The activation of NMDA receptors in the postsynaptic neuron starts the pathway, Ca^++^–activation of adenylate cyclase–activation of protein kinase A (PKA)–phosphorylation, and activation of CREB (cyclic-AMP response element-binding protein) induced transcription [48]. CaMKII kinase plays an important role in CREB phosphorylation. This event triggers the transcription of a group of genes that lead to protein synthesis and long-term synaptic plasticity [51].

Pathway analysis revealed interactions between the leucine-rich glioma-inactivated (LGI) protein family and ADAM proteins (a disintegrin and a metalloproteinase). The synapses’ formation and their maturation require the interaction between presynaptic and postsynaptic neurons, where different groups of synaptogenic proteins are engaged [52]. Another class of molecules that plays an important role in cellular interactions for the development and function of the nervous system is the LGI protein family. These are secreted synaptogenic proteins consisting of a leucine-rich repeat region and an epilepsy-related region (ΕΡΤΡ, epitempin) in humans [53]. Both protein domains are involved in protein–protein interactions. Genetic and biochemical evidence suggests that the mechanism of action of LGI proteins involves their binding to a subset of cell surface receptors belonging to the ADAM family (ADAM11, ADAM22, and ADAM23). These interactions play an important role in the development and function of the vertebrate nervous system, mediating synaptic transmission and myelination [53,54,55,56]. The LGI1 isoform is also a secretory protein interacting with ADAM family proteins. Although its mutations are associated with epilepsy in humans, its exact function in the CNS remains unclear. It appears to be involved in the regulation of signal transduction in stimulatory synapses and cerebellum development [57,58]. LGI1 binds simultaneously to the extracellular domains of the ADAM22 and ADAM23 proteins via the EPTP domain, enhancing and stabilizing excitatory synapses [57]. The association with ADAM23 regulates the “trimming” of dendrites and the effacement of synapses during development [50,51]. Accordingly, LGI1 and ADAM22 form a tripartite complex with the postsynaptic density protein 95 (PSD95). The latter is associated with the protein stargazin, a transmembrane regulatory subunit of the AMPA receptor, which is important for its trafficking and gating [58]. The association of LGI1 with ADAM23 and ADAM22 brings close pre- and postsynaptic membranes, stabilizing and enhancing neurotransmission [57]. The ADAM11 protein is essential for neuronal function itself. Mice deficient in ADAM11 had problems with learning, coordination of movements, and cognitive responses [59,60].

Finally, the LGI3 isoform is expressed in peripheral nerves and interacts with proteins ADAM22, ADAM 23, and syntaxin1 in the presynaptic complex, soluble NSF attachment protein/receptor (SNARE). In Pacific salmon (*Oncorhynchus keta*) brains, the upregulation of these genes has an important role in synaptic plasticity for olfactory imprinting and/or olfactory memory retrieval [61]. This protein has multiple roles, such as the induction of neurite growth and the phosphorylation of kinase B (AKT) and focal adhesion kinase (FAK) proteins, which control signal transduction [62]. It also promotes beta-amyloid and syntaxin1 endocytosis [63,64,65]. In our study, both syntaxin1 and nsfa transcripts were found upregulated in malpigmented fish.

According to our results, the transcripts encoding ion channels and, in particular, ion transfer via the “P” type ATPases are playing a significant role. “P” type ATPases is a large group that is evolutionarily associated with ion pumps found in bacteria, archaea, and eukaryotes. They seem to transform, remodel, and restructure between at least two configurations, E1 and E2. Many members of this family of proteins carry a wide range of cations [66]. The aforementioned ATPases affect ionic homeostasis, and they play an important role in cardiac function. Other types of ATPases are also present in the membrane of melanosomes, having an important role in pH setting. pH is a critical parameter for melanin synthesis. pH deregulation is associated with coloring defects in animals [40].

A process that appears to be different between normal and malpigmented juveniles is the process of keratinization. In mammals, the external layers of the mature epidermis keratinize; by contrast, in fish, the adult skin does not cornify. The fish skin is a mucosal epithelium, either in embryonic or adult stages, presenting a similar gene expression pattern [67]. Keratins are the most important structural proteins of the vertebrate epidermis and make up 85% of a fully differentiated keratinocyte. They belong to the protein superfamily of intermediate filaments, with a diameter of about 10 nm, consisting of α-helix spiral-wise dimers. Keratin fibrils are heteropolymers consisting essentially of type I and type II keratins [68]. Their expression patterns are related to the type of epithelium and the stage of differentiation. Keratin filaments run through the cytoplasm and bind to other membrane structures [69]. This reflects their main function of maintaining the mechanical stability of the cell and epithelial tissue [70]. Furthermore, differences were observed during the cornified envelope (CE) formation stages, particularly during the polymerization of the intermediate filaments of filaggrin (FLG) and keratin. This step facilitates the settling and widening of the cells on the outside of the epidermis and the formation of squamous [71,72]. Anomalies in the formation of the corneal envelope are associated with barrier dysfunction and structural defects in desmosome joints [73]. Responsible for these defects is p53 effector-related PMP-22 (PERP) protein, which is expressed during embryogenesis [74,75].

## 5. Conclusions

Conclusively, our results revealed that ambicolorated juveniles exhibit a divergent function mainly of the central nervous system at the synaptic level. In the present work, using high throughput sequencing techniques make it easier to compare holistically the expression patterns of biochemical and molecular networks and to identify critical differences in a short period of time during different stages of ontogenesis between the two above-mentioned categories [76]. The gene ontology enrichment analysis and the analysis carried out through the “Reactome” database demonstrated the major biochemical and molecular pathways that appear to be affected. The 157 identified gene sequences are mainly associated with ion transport, receptor internalization, synaptic vesicle endocytosis, membrane fusion, neuronal action potential, intermediate filament, cell junction, synapse, axon cytoplasm, voltage-gated sodium channel complex, presynapse, ionotropic glutamate receptor activity, extracellular-glutamate-gated ion channel activity, affecting crucial functions of the central nervous system, also the transport through ionic channels, muscular and cardiac function, keratinization, formation of the cornified envelope, ion homeostasis, CREB phosphorylation through the activation of CaMKII, Ras activation upon Ca^2+^ influx through NMDA receptor. Interestingly, the close association of pigmentation with genes that targeted the neurons and the nervous system at the gene expression level was documented; this was also reported previously in the scientific literature. The association of pigmentation, which occurs after embryogenesis, with the development of the nervous system, which occurs mainly in the early life stages, suggests that it is an organogenetic process, easily disrupted since the beginning of development. This means that it is necessary to further investigate the role of stress and rearing conditions in the early stages of life. Further studies should focus mainly on the role of ion channels and the neural synapses mentioned in the present work.

## Figures and Tables

**Table 1:** The most significant Reactome database paths detected based on the 157 identified transcripts.

Pathway Identifier		Pathway Name	FDR
1	R-DRE-438066	Unblocking of NMDA receptor, glutamate binding, and activation	2.81 × 10^−11^
2	R-DRE-936837	Ion transport by P-type ATPases	3.47 × 10^−10^
3	R-DRE-6805567	Keratinization	3.97 × 10^−10^
4	R-DRE-6809371	Formation of the cornified envelope	3.97 × 10^−10^
5	R-DRE-446107	Type I hemidesmosome assembly	4.83 × 10^−9^
6	R-DRE-5578775	Ion homeostasis	2.09 × 10^−8^
7	R-DRE-442755	Activation of NMDA receptor and postsynaptic events	4.01 × 10^−8^
8	R-DRE-399710	Activation of AMPA receptors	8.50 × 10^−7^
9	R-DRE-112314	Neurotransmitter receptors and postsynaptic signal transmission	8.50 × 10^−7^
10	R-DRE-416993	Trafficking of GluR2-containing AMPA receptors	3.49 × 10^−6^
11	R-DRE-399719	Trafficking of AMPA receptors	3.49 × 10^−6^
12	R-DRE-399721	Glutamate binding, activation of AMPA receptors, and synaptic plasticity	3.49 × 10^−6^
13	R-DRE-983712	Ion channel transport	3.86 × 10^−6^
14	R-DRE-112315	Transmission across Chemical Synapses	5.62 × 10^−6^
15	R-DRE-5576891	Cardiac conduction	2.24 × 10^−5^
16	R-DRE-442729	CREB phosphorylation through the activation of CaMKII	2.70 × 10^−5^
17	R-DRE-442982	Ras activation upon Ca^2+^ infux through NMDA receptor	3.53 × 10^−5^
18	R-DRE-397014	Muscle contraction	8.81 × 10^−5^
19	R-DRE-1266738	Developmental Biology	8.81 × 10^−5^
20	R-DRE-5682910	LGI-ADAM interactions	1.02 × 10^−4^

**Table 2:** The most significant categories of Gene ontology enrichment analysis based on the 157 d.e. transcripts.

Category	Term	Fisher Exact
GO_BIOLOGICAL_PROCESS	ion transport	2.90 × 10^−6^
	sodium ion transport	8.70 × 10^−5^
	receptor internalization	1.30 × 10^−4^
	synaptic vesicle endocytosis	2.60 × 10^−4^
	transport	3.70 × 10^−4^
	dynamin polymerization involved in mitochondrial fission	6.40 × 10^−4^
	membrane fusion	9.10 × 10^−4^
	neuronal action potential	1.50 × 10^−3^
	clathrin-mediated endocytosis	1.90 × 10^−3^
	calcium ion-regulated exocytosis of neurotransmitter	2.70 × 10^−3^
	regulation of exocytosis	3.80 × 10^−3^
	mitochondrial fission	4.30 × 10^−3^
	larval locomotory behavior	4.30 × 10^−3^
	dorsal/ventral pattern formation	6.20 × 10^−3^
	potassium ion transport	7.60 × 10^−3^
	microtubule cytoskeleton organization	8.10 × 10^−3^
	microtubule-based movement	1.60 × 10^−2^
GO_CELLULAR_COMPONENT	intermediate filament	6.20 × 10^−10^
	microtubule	2.50 × 10^−4^
	cell junction	2.50 × 10^−4^
	synapse	8.10 × 10^−4^
	sodium:potassium-exchanging ATPase complex	1.10 × 10^−3^
	axon cytoplasm	1.80 × 10^−3^
	voltage-gated sodium channel complex	3.90 × 10^−3^
	presynapse	6.10 × 10^−3^
	kinesin complex	9.10 × 10^−3^
	collagen trimer	1.30 × 10^−2^
	plasma membrane	2.40 × 10^−2^
GO_MOLECULAR_FUNCTION	structural molecule activity	5.40 × 10^−10^
	microtubule binding	3.80 × 10^−4^
	ionotropic glutamate receptor activity	7.40 × 10^−4^
	extracellular-glutamate-gated ion channel activity	8.30 × 10^−4^
	ATP-dependent microtubule motor activity, plus-end-directed	2.80 × 10^−3^
	voltage-gated sodium channel activity	2.80 × 10^−3^
	calcium-transporting ATPase activity	3.30 × 10^−3^
	ion channel activity	3.30 × 10^−3^
	clathrin binding	3.50 × 10^−3^
	sodium channel activity	3.80 × 10^−3^
	PDZ domain binding	4.30 × 10^−3^
	metalloendopeptidase activity	6.80 × 10^−3^
	receptor activity	8.90 × 10^−3^
	syntaxin binding	1.00 × 10^−2^
	metallopeptidase activity	1.10 × 10^−2^
	microtubule motor activity	1.50 × 10^−2^
	calcium ion binding	4.80 × 10^−2^

## Data Availability

Data available in a publicly accessible repository. The untreated raw data for the six created libraries were deposited in the NCBI Sequence Read Archive (study accession number PRJNA324631).

## Supplementary Materials

The following are available online at https://www.mdpi.com/article/10.3390/ani11082273/s1, Table S1: Differentially expressed transcripts (|log2FC| > 1 & FDR < 0.05).

## Abbreviations

ADAM	a disintegrin and metalloproteinase
AKT	protein kinase B
AMPA	α-amino-3-hydroxy-5-methyl-4-isoxazolepropionic acid
ARA	arachidonic acid
ATP	adenosine triphosphate
CaMKII	calmodulin-dependent protein kinase II
CE	cornified envelope
CEGMA	core eukaryotic genes mapping approach
CREB	cyclic-AMP response element-binding protein
DHA	docosahexaenoic acid
dph	days post-hatching
EPA	eicosapentaenoic acid
EPTP	epitempin
FAK	focal adhesion kinase
FDR	Benjamini–Hochberg false discovery rate
FLG	filaggrin
GDP	guanosine 5′-diphosphate
GluR1,2,3,4	glutamate receptor 1,2,3,4
GRIP	glutamate receptor interacting protein
GTP	guanosine 5′-triphosphate
hpf	hours post fertilization
LGI	leucine-rich glioma inactivated
LTP	long term potentiation
MCH	melanin-concentrating hormone
NCBI	national center for biotechnology information
NGS	next-generation sequencing
NMDA	N-methyl-D-aspartate
NSF	N-ethylmaleimide-sensitive fusion protein
PERP	p53 effector related to PMP-22
PICK	protein interacting with C kinase
PKC	protein kinase C
POMC	pro-opiomelanocortin
PSD95	postsynaptic density protein 95
SNAP	soluble NSF attachment protein
SNARE	soluble NSF attachment receptor
α-MSH	α-Melanocyte-stimulating hormone
