# Supplementary material for: Malpigmentation of Common Sole (Solea solea) during Metamorphosis Is Associated with Differential Synaptic-Related Gene Expression
Source: Animals (Basel). 2021 Aug 1;11(8):2273. doi: 10.3390/ani11082273 (PMC8388432; doi:10.3390/ani11082273)
Supplement: Supplementary file 1 [file animals-11-02273-s001.zip › animals-1260186-supplementary.pdf]

Table S1 Differentially expressed transcripts (|log_2FC| > 1 & FDR < 0.05).

A/A	ENSEMBL_GENE_ID	Name	Fold Change
1	ENSDARG00000062116	CTD (carboxy-terminal domain, RNA polymerase II, polypeptide A) small phosphatase-like b(ctdsplb)	2.67
2	ENSDARG00000057484	serine/arginine-rich splicing factor 2a(srsf2a)	2.07
3	ENSDARG00000101687	microtubule-associated protein 4-like(LOC100001114)	1.92
4	ENSDARG00000034817	N-acylsphingosine amidohydrolase (acid ceramidase) 1b(asah1b)	1.75
5	ENSDARG00000098936	kinesin family member 5A, a(kif5aa)	1.67
6	ENSDARG00000076679	retinoic acid induced 1(rai1)	1.59
7	ENSDARG00000054723	si:ch211-242b18.1(si:ch211-242b18.1)	1.57
8	ENSDARG00000067964	solute carrier family 6 (neurotransmitter transporter), member 5(slc6a5)	1.56
9	ENSDARG00000063133	solute carrier family 4, NaHCO3 transporter, member 10a(slc4a10a)	1.53
10	ENSDARG00000058558	leucine zipper, putative tumor suppressor family member 3b(lzts3b)	1.52
11	ENSDARG00000045316	MAP7 domain containing 2b(map7d2b)	1.51
12	ENSDARG00000060434	microtubule-associated protein 1B(map1b)	1.47
13	ENSDARG00000061647	neurexin 1a(nrxn1a)	1.47
14	ENSDARG00000005775	sodium channel, voltage gated, type VIII, alpha subunit a(scn8aa)	1.45
15	ENSDARG00000042014	cytochrome P450, family 11, subfamily C, polypeptide 1(cyp11c1)	1.45
16	ENSDARG00000052765	glutamate receptor, ionotropic, AMPA 2b(gria2b)	1.45
17	ENSDARG00000056206	Ca/calmodulin-dependent prot. kin. (CaM kinase) II gamma 2(camk2g2)	1.45
18	ENSDARG00000042837	ATPase, Na+/K+ transporting, beta 3b polypeptide(atp1b3b)	1.44
19	ENSDARG00000018259	ATPase, Na+/K+ transporting, alpha 3a polypeptide(atp1a3a)	1.43
20	ENSDARG00000100089	calcium/calmodulin-dep. prot. kinase (CaM kinase) II beta 2 (camk2b2)	1.42
21	ENSDARG00000057016	cell division cycle 14Ab(cdc14ab)	1.40
22	ENSDARG00000103435	sorbin and SH3 domain containing 1 (sorbs1)	1.40
23	ENSDARG00000097576	zgc:92912(zgc:92912)	1.38
24	ENSDARG00000002031	TOX high mobility group box family member 4 a(tox4a)	1.37
25	ENSDARG00000068989	gamma-aminobutyric acid (GABA) A receptor, alpha 1 (gabra1)	1.37
26	ENSDARG00000023445	ATPase, Ca++ transporting, plasma membrane 3b(atp2b3b)	1.37
27	ENSDARG00000027828	glutamate receptor, ionotropic, N-methyl D-aspartate 1a(grin1a)	1.35
28	ENSDARG00000030614	synaptotagmin Ia(syt1a)	1.35
29	ENSDARG00000025206	synaptotagmin IIa(syt2a)	1.35
30	ENSDARG00000079204	ADAM metallopeptidase domain 11(adam11)	1.35
31	ENSDARG00000061918	complexin 2(cplx2)	1.35
32	ENSDARG00000069265	matrilin 3b(matn3b)	1.34
33	ENSDARG00000090872	si:dkey-276j7.1(si:dkey-276j7.1)	1.32
34	ENSDARG00000010052	ndrg family member 3b(ndrg3b)	1.32
35	ENSDARG00000023228	visinin-like 1a(vsnl1a)	1.32
36	ENSDARG00000104401	YME1-like 1b(yme1l1b)	1.32
37	ENSDARG00000007654	N-ethylmaleimide-sensitive factor a(nsfa)	1.31

38	ENSDARG00000059596	CAP-GLY domain containing linker protein 2(clip2)	1.31
39	ENSDARG00000056910	neurofascin homolog (chicken) b(nfascb)	1.31
40	ENSDARG00000105490	rabphilin 3A homolog (mouse), b(rph3ab)	1.30
41	ENSDARG00000015931	si:ch211-214j24.9(si:ch211-214j24.9)	1.30
42	ENSDARG00000099487	si:ch73-233f7.7 (PCDHGC3)	1.30
43	ENSDARG00000100851	gephyrin b(gphnb)	1.29
44	ENSDARG00000053201	zgc:172323(zgc:172323)	1.29
45	ENSDARG00000060532	ADAM metallopeptidase domain 22(adam22)	1.28
46	ENSDARG00000014792	leucine rich repeat containing 4Bb(lrrc4bb)	1.28
47	ENSDARG00000061817	kinesin family member 1Aa(kif1aa)	1.28
48	ENSDARG00000027963	CaM kinase-like vesicle-associated a(camkva)	1.27
49	ENSDARG00000105083	chromodomain helicase DNA binding protein 5(chd5)	1.27
50	ENSDARG00000025301	glial fibrillary acidic protein(gfap)	1.25
51	ENSDARG00000103841	Chromosome 25: 23,392,431-23,397,900 (CU024870.1)	1.25
52	ENSDARG00000059601	microtubule-associated protein 1Aa(map1aa)	1.25
53	ENSDARG00000104945	synuclein, beta(sncb)	1.25
54	ENSDARG00000010042	dynamin 1a(dnm1a)	1.24
55	ENSDARG00000074866	protein tyrosine phosphatase, non-receptor type 5(ptpn5)	1.24
56	ENSDARG00000074524	contactin associated protein 1(cntnap1)	1.24
57	ENSDARG00000067509	solute carrier family 24 (Na/K/Ca exchanger), member 4b(slc24a4b)	1.22
58	ENSDARG00000069484	DAB2 interacting protein a(dab2ipa)	1.22
59	ENSDARG00000018032	sodium channel, voltage gated, type VIII, alpha subunit b(scn8ab)	1.22
60	ENSDARG00000036584	ST8 alpha-N-acetyl-neuraminide alpha-2,8-sialyltransferase 5(st8sia5)	1.22
61	ENSDARG00000076027	kinesin family member 5C(kif5c)	1.22
62	ENSDARG00000021351	neurofilament, medium polypeptide a(nefma)	1.21
63	ENSDARG00000091783	si:dkeyp-71f10.5	1.21
64	ENSDARG00000068589	vimentin(LOC572200)	1.21
65	ENSDARG00000002970	synovial sarcoma translocation, chromosome 18 (H. sapiens)(ss18)	1.21
66	ENSDARG00000038574	secretogranin II (chromogranin C), b(scg2b)	1.20
67	ENSDARG00000015174	ATPase, H+ transporting, lysosomal V0 subunit a1b(atp6v0a1b)	1.20
68	ENSDARG00000007824	calmodulin binding transcription activator 1b(camta1b)	1.19
69	ENSDARG00000102702	leucine-rich repeat neuronal protein 3-like (LOC100535105)	1.19
70	ENSDARG00000074745	zmp:0000000760(zmp:0000000760)	1.18
71	ENSDARG00000020609	synaptosomal-associated protein, 25a(snap25a)	1.18
72	ENSDARG00000097528	si:dkey-7j14.5(si:dkey-7j14.5)	1.17
73	ENSDARG00000070173	glutamate receptor, ionotropic, AMPA 2a(gria2a)	1.16
74	ENSDARG00000102690	regulating synaptic membrane exocytosis 2b(rims2b)	1.16
75	ENSDARG00000105188	si:dkey-76i15.1(si:dkey-76i15.1)	1.16
76	ENSDARG00000027740	adenylate cyclase activating polypeptide 1b(adcyap1b)	1.15
77	ENSDARG00000009281	dynamin 1b(dnm1b)	1.15
78	ENSDARG00000099203	ATPase, Na+/K+ transporting, beta 2a polypeptide(atp1b2a)	1.15

79	ENSDARG00000000503	syntaxin 1B(stx1b)	1.14
80	ENSDARG00000057317	nexilin (F actin binding protein)(nexn)	1.14
81	ENSDARG00000062096	peptidase M20 domain containing 1, tandem duplicate 2(pm20d1.2)	1.11
82	ENSDARG00000062531	mitogen-activated protein kinase 8 interacting protein 3(mapk8ip3)	1.11
83	ENSDARG00000102009	protein phosphatase 2, regulatory subunit B, delta (ppp2r2d)	1.09
84	ENSDARG00000104937	protein tyrosine phosphatase, receptor type D(ptprd)	1.08
85	ENSDARG00000015554	zic family member 2 (odd-paired homolog, Drosophila), a(zic2a)	1.07
86	ENSDARG00000014232	si:dkey-121j17.5	1.06
87	ENSDARG00000029692	RUN and FYVE domain containing 3(rufy3)	1.05
88	ENSDARG00000042948	DnaJ (Hsp40) homolog, subfamily C, member 5aa(dnajc5aa)	1.04
89	ENSDARG00000087247	K voltage-gated channel, shaker-related subfamily, beta member 2 a(kcnab2a)	1.04
90	ENSDARG00000074561	si:dkey-175a17.3(si:dkey-175a17.3)	1.02
91	ENSDARG00000102558	phosphodiesterase 6H, cGMP-specific, cone, gamma(pde6h)	1.01
92	ENSDARG00000034215	RAB42, member RAS oncogene family a(rab42a)	1.01
93	ENSDARG00000063433	ATPase, Ca++ transporting, plasma membrane 2(atp2b2)	1.01
94	ENSDARG00000055455	glycoprotein M6Aa(gpm6aa)	1.01
95	ENSDARG00000061436	collagen, type VI, alpha 2(col6a2)	−1.00
96	ENSDARG00000005762	collagen, type XIV, alpha 1a(col14a1a)	−1.00
97	ENSDARG00000006456	platelet-derived growth factor receptor-like(pdgfrl)	−1.01
98	ENSDARG00000040628	zgc:110333(zgc:110333)	−1.01
99	ENSDARG00000004527	protein (peptidylprolyl cis/trans isomerase) NIMA-interacting, 4 (parvulin)(pin4)	−1.03
100	ENSDARG00000090009	si:ch73-167i17.7	−1.03
101	ENSDARG00000092731	major histocompatibility complex class I UKA(mhc1uka)	−1.03
102	ENSDARG00000004472	alpha tubulin acetyltransferase 1(atat1)	−1.04
103	ENSDARG00000042245	myosin, light chain 13(myl13)	−1.04
104	ENSDARG00000062750	si:ch73-74h11.1(si:ch73-74h11.1)	−1.05
105	ENSDARG00000078018	si:dkey-79d12.4(si:dkey-79d12.4)	−1.05
106	ENSDARG00000100753	CD36 antigen-like(LOC564077)	−1.06
107	ENSDARG00000100573	si:ch211-255a21.1(si:ch211-255a21.1)	−1.06
108	ENSDARG00000069888	claudin a(cldna)	−1.06
109	ENSDARG00000075015	heme-binding protein soul5(soul5)	−1.07
110	ENSDARG00000027984	glutathione S-transferase zeta 1(gstz1)	−1.07
111	ENSDARG00000057571	phosphoglycerate mutase 2 (muscle)(pgam2)	−1.07
112	ENSDARG00000060345	apolipoprotein Da, duplicate 1(apoda.1)	−1.07
113	ENSDARG00000099947	Scaffold KN149855.1: 6,271-9,010 (CABZ01109019.1)	−1.08
114	ENSDARG00000036832	type I cytokeratin, enveloping layer, like(cyt1l)	−1.10
115	ENSDARG00000036086	integrin, alpha 11a (itga11a)	−1.14
116	ENSDARG00000041430	bone morphogenetic protein 2b(bmp2b)	−1.15
117	ENSDARG00000069518	aquaporin 3b(aqp3b)	−1.15

118	ENSDARG00000057590	si:ch1073-396h14.1(si:ch1073-396h14.1)	−1.15
119	ENSDARG00000100450	bloodthirsty-related gene family, member 21(btr21)	−1.16
120	ENSDARG00000097539	si:ch211-39f2.3(si:ch211-39f2.3)	−1.16
121	ENSDARG00000105182	N-terminal asparagine amidase (ntan1)	−1.17
122	ENSDARG00000077082	angiotensin II receptor-associated protein(agtrap)	−1.17
123	ENSDARG00000076321	collagen type XXVIII alpha 1 a(col28a1a)	−1.17
124	ENSDARG00000045676	calumenin a(calua)	−1.19
125	ENSDARG00000036834	keratin 92(krt92)	−1.20
126	ENSDARG00000036830	keratin 91(krt91)	−1.20
127	ENSDARG00000053476	lipase, hepatic a(lipca)	−1.21
128	ENSDARG00000056178	dermatopontin(dpt)	−1.22
129	ENSDARG00000073764	si:ch211-113j14.1(si:ch211-113j14.1)	−1.22
130	ENSDARG00000057575	purine nucleoside phosphorylase 4a(pnp4a)	−1.22
131	ENSDARG00000099399	thyroid hormone responsive(thrsp)	−1.23
132	ENSDARG00000098203	APC down-regulated 1 (APCDD1)	−1.25
133	ENSDARG00000102415	scinderin like a(scinla)	−1.26
134	ENSDARG00000015059	dishevelled associated activator of morphogenesis 1a(daam1a)	−1.27
135	ENSDARG00000094752	retinal pigment epithelium-specific protein 65b(rpe65b)	−1.27
136	ENSDARG00000035329	calpain, small subunit 1 a(capns1a)	−1.27
137	ENSDARG00000067727	im:7142702(im:7142702)	−1.30
138	ENSDARG00000004358	guanine nucleotide bind. prot. (G protein), beta polypeptide 3a(gnb3a)	−1.31
139	ENSDARG00000103034	cysteine-rich venom protein natrin-1-like(LOC100536500)	−1.33
140	ENSDARG00000034670	ubiquitin-conjugating enzyme E2E 2(ube2e2)	−1.36
141	ENSDARG00000077721	lysine-rich nucleolar protein 1(knop1)	−1.40
142	ENSDARG00000029524	IMP (inosine 5'-monophosphate) dehydrogenase 1b(impdh1b)	−1.40
143	ENSDARG00000104771	translocase of inner mitochondrial membrane 44 homolog (yeast)(timm44)	−1.45
144	ENSDARG00000011956	distal-less homeobox 4a(dlx4a)	−1.46
145	ENSDARG00000101535	collagen, type X, alpha 1b(col10a1b)	−1.50
146	ENSDARG00000062494	olfactomedin-like 1(olfml1)	−1.52
147	ENSDARG00000028542	si:ch211-114c12.2(si:ch211-114c12.2)	−1.53
148	ENSDARG00000104267	periostin, osteoblast specific factor b(postnb)	−1.55
149	ENSDARG00000078859	G0/G1 switch 2(g0s2)	−1.60
150	ENSDARG00000017314	zgc:92041(zgc:92041)	−1.63
151	ENSDARG00000003938	replication protein A1(rpa1)	−1.74
152	ENSDARG00000092947	type I cytokeratin, enveloping layer(cyt1)	−1.87
153	ENSDARG00000058323	transmembrane BAX inhibitor motif containing 1(tmbim1)	−1.92
154	ENSDARG00000001676	glycoprotein M6Bb(gpm6bb)	−2.69
155	ENSDARG00000098344	RAB18B, member RAS oncogene family(rab18b)	−2.78
156	ENSDARG00000035338	tubulin polymerization-promoting protein family member 2(tppp2)	−2.86
157	ENSDARG00000078416	zinc finger E-box binding homeobox 2b(zeb2b)	−2.93
